# Impact of Second Trimester Maternal Dietary Intake on Gestational Weight Gain and Neonatal Birth Weight

**DOI:** 10.3390/nu9060627

**Published:** 2017-06-17

**Authors:** Malshani L. Pathirathna, Kayoko Sekijima, Mieko Sadakata, Naoshi Fujiwara, Yoshiyuki Muramatsu, Kuruppu M.S. Wimalasiri

**Affiliations:** 1 Department of Nursing, Graduate School of Health Sciences, Niigata University, 2-746 Asahimachi-dori, Chuo-ku, Niigata 951-8518, Japan; sekijima@clg.niigata-u.ac.jp (K.S.); atom@clg.niigata-u.ac.jp (M.S.); murayosi@clg.niigata-u.ac.jp (Y.M.); 2 Department of Nursing, Faculty of Allied Health Sciences, University of Peradeniya, Peradeniya 20400, Sri Lanka; 3 Department of Medical Technology, Graduate School of Health Sciences, Niigata University, 2-746 Asahimachi-dori, Chuo-ku, Niigata 951-8518, Japan; fujiwaranaoshi@gmail.com; 4 Department of Food Science and Technology, Faculty of Agriculture, University of Peradeniya, Peradeniya 20400, Sri Lanka; swarnaw@pdn.ac.lk

**Keywords:** low birth weight, maternal diet, macronutrient, gestational weight gain, nutrition, Sri Lanka

## Abstract

Poor maternal nutrition is a major contributor to the high incidence of low birth weight deliveries in developing countries. This study aimed to assess the impact of second trimester maternal dietary intake on gestational weight gain and neonatal birth weight. A longitudinal study was conducted in a tertiary care hospital in Sri Lanka. Participants were 141 pregnant women at 18–24 weeks gestation who were followed up until delivery. Maternal dietary intake was assessed using a validated Food Frequency Questionnaire at 21.1 ± 1.8 gestational weeks. Gestational weight gain was examined at the end of 28 weeks gestation and at the end of pregnancy. Energy and nutrient intakes were calculated using NutriSurvey 2007 (EBISpro, Willstaett, Germany) nutrient analysis software, modified for Sri Lankan foods. The mean total gestational weight gain of women with low carbohydrate intake (229–429 g/day) was 2.2 kg less than that of women with moderate carbohydrate intake (430–629 g/day) (95% confidence interval (CI) 0.428–4.083 kg; *p* = 0.016). Similarly, babies of women with low carbohydrate intake were 312 g lighter compared with those of women with a moderate carbohydrate intake (95% CI 91–534 g; *p* = 0.006). Our results suggest that second trimester maternal carbohydrate intake has significant impacts on total gestational weight gain and neonatal birth weight.

## 1. Introduction

Despite a consistent decline in maternal and infant mortality, Sri Lanka continues to experience crucial health problems among pregnant women, infants, and children. A significant problem is the high percentage of low birth weight (LBW) deliveries. LBW is defined as weight at birth less than 2500 g regardless of gestational age. According to 2012 national health statistics, there were 55,557 LBW deliveries in Sri Lanka, accounting for 16.3% of total live births [1].

Perinatal complications associated with LBW are mostly attributed to fetal prematurity, but may also result from intrauterine growth restriction [2]. LBW increases the risk of infant mortality [3], infectious diseases, inhibited growth, and inhibited cognitive development. Children with LBW are also more likely to suffer from chronic illnesses in later life [4]. When overall LBW rates are examined, Sri Lanka performs better than other countries in the region that have a similar income level. However, multiple causative factors are associated with Sri Lanka’s low mean birth weight, including preterm deliveries, pregnancy complications, and increased interest in elective cesarean sections before 40 weeks gestation.

Maternal malnutrition is the main contributing factor underlying high LBW percentages in many developing countries [5], and plays a major role in both maternal and child health. Poor maternal nutrition has been related to adverse pregnancy outcomes. However, this association is complex, influenced by many intrinsic and extrinsic factors, and often results from socio-cultural and behavioral factors. Body mass index (BMI) and gestational weight gain (GWG) are indicators of maternal nutrition. Understanding the association between maternal nutrition and neonatal birth weight may inform development of nutritional interventions that improve neonatal birth weight to within normal limits, improve long-term quality of life, and reduce the healthcare burden. Early fetal nutrition is considered the most important factor in child health, even before birth. If a woman is undernourished in early pregnancy, the fetal metabolism will be altered as fetal adaption to the undernourished utero-placental environment takes place. Ultimately, this slows the overall fetal growth rate and increases the risk for LBW. However, there are lack of human studies that clarify the best timing for optimum nutrition during early pregnancy. In addition, during the first trimester of pregnancy, almost all women experience loss of appetite, nausea and vomiting, making it difficult to obtain sufficient nutrition. Therefore, second trimester maternal nutrition can be considered as an important turning point for both the mother and fetus.

Sri Lankans have a unique dietary pattern. The staple meal is large serving of rice accompanied by different vegetable, egg, meat or fish side dishes cooked with spices and (most often) coconut milk. This rice and curry meal is commonly consumed as lunch, although it may also form breakfast and dinner, depending on personal preferences and factors such as economic status. Traditional morning and evening meals usually comprise a starchy staple such as *string hoppers, hoppers, pittu* or bread with one or two curries. To date, no studies on the relationships among maternal dietary intake, gestational weight gain, and neonatal birth weight have been conducted in Sri Lanka. This study aimed to assess the influence of second trimester maternal dietary intake on GWG and neonatal birth weight, and to explore the relationships among these three factors.

## 2. Materials and Methods

### 2.1. Design, Setting and Participants

This study used a longitudinal design, and was conducted in antenatal clinics at the General Hospital Kurunegala, Sri Lanka from October 2015 to June 2016. A convenience sample of pregnant women at 18–24 weeks gestation was included and they were followed up until delivery. Initial recruitment consisted of 150 pregnant women.

### 2.2. Procedure

Women were invited to participate in the study through a recruitment poster displayed in the hospital’s outpatient department. Pregnant women who were interested in participating were requested to inform the researcher directly, in person or via telephone. Written information sheets were provided to all interested women, including an explanation of the study purpose, data collection methods, time taken for data collection and confidentiality of personal information.

Exclusion criteria were risk factors according to obstetric history (e.g., miscarriages/abortions, multiple fetuses, pregnancy-induced hypertension, and gestational diabetes mellitus) and medical history (e.g., psychiatric disorder or long term cardiac, renal, lung or gastrointestinal disease). Impending neonates with a 5-minute Apgar score less than 5, and women who expected to deliver their baby at another hospital were also excluded. Based on these criteria, there were seven exclusions by the researcher (spontaneous abortion *n* = 2, multiple fetuses identified at the 20-week ultra sound scan *n* = 2, and maternal desire to deliver at another hospital *n* = 3) and two personal withdrawals, resulting in nine exclusions between the recruitment process and the end of pregnancy.

Neonatal data were collected from hospital records after participating women had delivered their babies. The researcher failed to find neonatal data for 14 neonates because of confusion with hospital delivery registry records, as many similar names made it difficult to accurately locate study participants. One neonate with a 5-minute Apgar score less than 5 was excluded at the end of data collection. Data were collected for 126 neonates (Figure S1).

Socioeconomic and demographic data were collected by the researcher through interviews using a pre-designed and pre-tested questionnaire. Maternal body weight and height were measured using standard scales. Pre-pregnancy BMI was calculated by pre-pregnancy weight in kg (weight at the first antenatal clinic visit, usually 6–8 weeks gestation) divided by height in m^2^. Women’s weight at their first antenatal clinic visit was obtained from individual pregnancy cards. Height was measured at the time of study recruitment. World Health Organization (WHO) international BMI cut-off points were used for BMI group categorization [6]. The Institute of Medicine (IOM) 2009 Re-examined Guidelines [7] were used to define total GWG categories: 12.5–18 kg for underweight women, 11.5–16 kg for women of normal weight, 7–11 kg for overweight women, and 5–9 kg for obese women. Maternal GWG was measured at two points: at the end of 28 weeks gestation and at the end of pregnancy. GWG up to 28 weeks gestation was calculated by taking the positive difference between the pre-pregnancy weight and that measured at 28 weeks gestation. Total GWG was calculated by subtracting pre-pregnancy weight from delivery weight (38.8 ± 1.5 gestational weeks). Delivery weight was obtained from each woman’s hospital records.

Participants’ dietary intake was assessed using a Food Frequency Questionnaire (FFQ) that was developed and validated for Sri Lankan adults, including women [8,9]. *Thriposha*, a supplement of pulses provided to pregnant women, was included in the FFQ under the pulses group. This is a nutrient supplement pack that is given to all pregnant women through community maternity clinics to combat protein, energy, and micronutrient deficiencies. It is a pre-cooked and ready-to-eat cereal legume-based food. In total, 100 g of *Thriposha* contains 401.8 kcal of energy, 61.9 g of carbohydrate, 20.0 g of protein, 7.8 g of fat, 1700 IU of vitamin A, and 18 mg of iron. It is recommended to consume 50 g of *Thriposha* each day during pregnancy. Participants were asked to complete the FFQ once in the second trimester (21.1 ± 1.8 gestational weeks). The FFQ was self-administered, with support from the researcher provided where necessary. Questions focused on the women’s usual dietary intake in the second trimester. Food photographs were included in the questionnaire to facilitate understanding of portion sizes. Dietary assessment aids were day-to-day standard household measurements (e.g., plate, cup, glass and spoons of different sizes). Energy and nutrient intakes were calculated using NutriSurvey 2007 (EBISpro, Willstaett, Germany) nutrient analysis software, after modification for individual Sri Lankan food items and recipes.

#### 2.2.1. Nutrient Analysis Software Modification for Sri Lankan Food

Modification of the nutrient analysis software involved adding single food items to the original software using information from the food composition tables of Sri Lanka [10], and the United States Department of Agriculture (USDA) nutrient database [11]. It should be noted there is no updated Sri Lankan nutritional database up to date. Macronutrient and micronutrient values for single food items in the original software and those from the USDA database were changed to reflect local food using the food composition tables of Sri Lanka. Available nutrient data from food packaging were used for local food items such as processed foods, cookies and snacks. A standard local recipe book was used for curry/mixed dishes [12]. All recipes were checked for validity by consulting a convenience sample of 10 participants from the study group, and the recipes were modified accordingly. To estimate portion sizes for curry/mixed dishes, a procedure was adapted from a previous study [13] (Figure S2):

- For curry/mixed dishes, ingredients were weighed to the nearest 1 g of edible portion using a standard kitchen weighing scale (Tanita, no. 1155).
- Dishes were cooked according to the validated recipes.
- The final products were measured using standard household measurement utensils.

Recipes (curry/mixed dishes) were entered in to the modified software by entering single food items according to the validated recipes. For each recipe, a cooking method was applied from the available options in the software to approximate weight loss due to water evaporation from different food preparation methods. The software then automatically calculated and standardized the nutrient composition for 100 g of final product. One cup was considered 150 mL and one glass 200 mL. The portion size of each food item in the FFQ was also measured and the software was modified as necessary. After the final software modification, the FFQ was entered in the software with the option of changing frequency of consumption to daily, weekly or monthly. Finally, participants’ FFQ data were entered into the software and estimated energy, carbohydrate, and protein intakes per day were calculated.

#### 2.2.2. Calculation of Estimated Energy Requirement (EER)

The EER for the second trimester of pregnancy for each woman was calculated based on IOM guidelines [14]:

EER (second trimester) = non-pregnant EER + 340 kcal(1)

EER (non-pregnant) = 354 − (6.91 × age (years)) + physical activity coefficient × (9.36 × weight (kg) + 726 × height (m))(2)

The value of active physical level (1.27) for women aged 19 years and older [14] was used as the physical activity coefficient. All the pregnant women reported that they were engaging in normal day-to-day activities and there were no women with prescribed activity limitations.

#### 2.2.3. Recommended Dietary Allowance (RDA) of Protein

The RDA of protein during the second trimester for each woman was calculated as 1.1 g/kg/day, using the women’s pre-pregnancy weight [15].

### 2.3. Data Analysis

All data were entered and rechecked in Microsoft Excel 2007. Data for energy and macronutrient intakes were transferred from NutriSurvey 2007 to Minitab version 17 for statistical analysis. Descriptive statistics were expressed as mean ± standard deviation (SD). Correlations between neonatal birth weight, gestational weight gain, dietary intake and other continuous variables were evaluated with Pearson’s correlation analysis. To test the effects of different levels of energy and macronutrient intakes on gestational weight gain and neonatal birth weight, participating women were divided into two groups by energy intake (1 = daily energy intake less than the EER; 2 = daily energy intake greater than or equal to EER) and two protein intake groups (1 = less than the RDA of protein intake; 2 = greater than or equal to RDA of protein intake) based on estimated dietary intakes. As all women were above the RDA for carbohydrate, they were grouped in three categories with same class interval: 1 = 229–429 g/day; 2 = 430–629 g/day; and 3 = 630–829 g/day. One-way analysis of variance (ANOVA) was used to examine the differences in means of gestational weight gain, energy and nutrient intakes and neonatal birth weight. Two general linear models were constructed to define independent factors associated with total gestational weight gain and neonatal birth weight, controlling for confounders. Total energy intake was not included in the general linear models to avoid multicollinearity, as the total energy intake represents energy from carbohydrate, protein, and fat. For each analysis, 95% confidence intervals were calculated, and *p* < 0.05 was considered statistically significant.

### 2.4. Ethics

Informed written consent was obtained from all the subjects before they participated in the study. The study was conducted in compliance with the principles of the Declaration of Helsinki, and the protocol was approved by the Ethical Review Committee, Graduate School of Health Sciences, Niigata University, Japan (No: 125); the Ethical Review Committee, Faculty of Allied Health Sciences, University of Peradeniya, Sri Lanka; and the Institutional Ethical Review Committee, Teaching Hospital Kurunegala, Sri Lanka (No: ERC/2015/06).

## 3. Results

### 3.1. Participants’Characteristics

The final sample included 126 healthy newborns. Of these, 22 (17.4%) were LBW babies. In total, 20.6% women were underweight when they became pregnant and 56.7% had normal BMI. Participants’ characteristics are summarized in Table 1.

Of the 141 pregnant women, 138 returned a completed FFQ. Two incomplete questionnaires were excluded from the final analysis. The mean EER was 2224.4 ± 138.6 kcal/day; 26 (19.1%) participants were below the EER, whereas almost all women (100%) had a carbohydrate intake above the RDA (equal to 175 g/day). The mean RDA of protein was 57.3 ± 11.3 g/day, and 81.6% women had a protein intake above the RDA (calculated on an individual basis). Of the total energy intake, 72.8% was from carbohydrate and 9.8% was from protein. The protein intake mainly comprised plant protein (75.7% of total protein intake), with only 12.8% of the total protein intake being animal protein. The mean animal protein intake was 9.1 g/day and the mean plant protein was intake 53.9 g/day. Women with the lowest level of monthly household income showed the lowest total energy, carbohydrate, and protein intakes, but this did not reach statistical significance. Women living in rural areas showed the highest carbohydrate intake, but this did not significantly differ from women in urban and sub-urban areas (Table S1).

### 3.2. Correlation among Maternal Parameters, GWG and Neonatal Birth Weight

The correlation between total GWG and neonatal birth weight was 0.194 (*p* = 0.046). When the analysis was repeated with pre-pregnancy BMI and gestational age fixed, there was a moderate positive correlation between total GWG and neonatal birth weight (*r* = 0.302, *p* = 0.002). Maternal height (*r* = 0.283), pre-pregnancy weight (*r* = −0.247), and pre-pregnancy BMI (*r* = −0.340) were significantly correlated with total GWG (*p* < 0.05). Gestational age (*r* = 0.315), pre-pregnancy weight (*r* = 0.234), and pre-pregnancy BMI (*r* = 0.187) were significantly correlated with neonatal birth weight (*p* < 0.05). A strong positive correlation was observed between GWG up to 28 weeks gestation and total GWG (*r* = 0.812, *p* < 0.001). None of the studied dietary factors (total energy, carbohydrate, protein, and fat intakes) showed a significant correlation with total GWG or neonatal birth weight.

### 3.3. Effects of Maternal and Neonatal Characteristics on GWG

A univariate analysis was performed to test the effects of maternal and neonatal characteristics on GWG. Of the factors assessed, only the pre-pregnancy BMI category showed a significant relationship with GWG up to 28 weeks gestation (*p* < 0.001), whereas both the pre-pregnancy BMI category (*p* < 0.001) and dietary protein intake category (*p* < 0.05) were significantly associated with total GWG (Table 2).

The fitted general linear model (Table 3) showed significant effects of education level (*p* < 0.05), pre-pregnancy BMI category (*p* < 0.001), and daily carbohydrate intake category (*p* < 0.05) on total GWG. The respective effects of fat intake, parity, and dietary protein intake category were not significant (*p* > 0.05). Women with an underweight pre-pregnancy BMI showed a higher mean total GWG (3.8 kg) compared with women with normal pre-pregnancy BMI (*p* < 0.001). Similarly, women with underweight pre-pregnancy BMI showed a 4.8 kg higher mean total GWG compared with women who were overweight (*p* < 0.001) and a 5.4 kg higher gain than those who were obese (*p* < 0.001). The mean total GWG for women with a carbohydrate intake of 229–429 kcal/day was 2.2 kg below that of women with a carbohydrate intake of 430–629 kcal/day (*p* = 0.016).

### 3.4. Effects of Maternal and Neonatal Characteristics on Neonatal Birth Weight

The univariate analysis revealed that five of 12 factors (area of residence, history of LBW delivery, total GWG category, total energy intake category, and carbohydrate intake category) were significantly associated with neonatal birth weight (*p* < 0.05) (Table 4).

The general linear model (*R^2^* (adjusted) = 13.3%) showed that pre-pregnancy BMI (*p* = 0.025), gestational age (*p* < 0.001), parity (*p* < 0.05), and carbohydrate intake category (*p* < 0.05) had significant effects on neonatal birth weight. The babies of urban mothers were 258 g lighter than those of rural mothers; However, the difference was not significant (*p* = 0.062). The mean birth weight of babies of primiparous mothers was 187.4 g below that of babies of multiparous mothers (*p* = 0.046). For the carbohydrate intake category, the mean neonatal birth weight for category 1 (229–429 kcal/day) was 312 g below the mean birth weight of those in category 2 (430–629 kcal/day) (*p* = 0.006). Income level had no significant effect on neonatal birth weight (*p* > 0.05) (Table 5).

### 3.5. Effects of Supplemental Foods on GWG and Neonatal Birth Weight

The mean *Thriposha* intake was 34.8 ± 29.3 g/day (range 0–200 g/day); 11.8% (16/136) of women reported they were not consuming the supplement at all. There was no significant difference in total GWG between women who consumed 0–49 g/day of *Thriposha* (9.0 ± 3.7 kg) and those who consumed ≥50 g/day (9.8 ± 3.9 kg) (*p* = 0.271). The mean neonatal birth weight was 2859.1 ± 488.4 g in mothers who consumed 0–49 g/day of *Thriposha*, and 2906.3 ± 523.1 g in those who consumed ≥50 g/day. There was no significant difference in mean neonatal birth weight between the two *Thriposha* groups (F = 0.26, *p* = 0.608).

## 4. Discussion

The present study showed that 17.4% of deliveries were LBW babies, which was slightly above the national LBW rates for 2014 [16]. This might be because data for the present study were collected in a large tertiary care hospital that included more complicated pregnancies. In the present study, the average total GWG was 9.3 ± 3.7 kg, which was slightly below that observed for Sri Lankan women in a previous study [17]. However, this mean total GWG was below that recommended for underweight and normal BMI women. We found a moderate positive correlation between total GWG and neonatal birth weight. Therefore, total GWG may be considered a good predictor of neonatal birth weight, this emphasizes the importance of appropriate GWG when it is sought to prevent LBW deliveries. As there was a strong positive correlation between total GWG and GWG up to 28 weeks gestation, GWG up to 28 weeks can be used as a meaningful predictor to optimize individualized antenatal care for women who show low weight gain. Pre-pregnancy BMI also showed a significant association with neonatal birth weight, indicating the importance of maternal nutrition at the time a woman becomes pregnant.

In the present study, all participating women had a carbohydrate intake during pregnancy above the RDA. The mean energy intake was significantly higher than previous studies [18,19]. However, consuming a large serving of rice and/or other starchy staple in all three main meals and the daily consumption of supplemental food with a higher energy value are central to this high energy intake. In addition, the concept of “eating for two” during pregnancy still prevails in Sri Lanka, even though it has no scientific basis. In the present study, 18.4% of women had a protein intake during pregnancy below the RDA, with main protein supply being plant protein. Although protein from animal sources is of greater nutritional value because it contains all essential amino acids, the animal protein intake of women in our sample was low compared with Western countries [20]. This may be explained by the vegetarian trend of the younger generation in Sri Lanka because of religious and cultural influences and the poor economic status. There is no separate RDA for fat intake during pregnancy, meaning that fat is recommended to be 25–35% of the total calorie intake, as for the general population. Therefore, in an average 2200 kcal diet, approximately 550 kcal should be fat (approximately 60 g). The present study showed a mean fat intake of 45.8 ± 16.9 g per day, which was moderately below the general recommendation.

Our study revealed a significant relationship between second trimester carbohydrate intake and neonatal birth weight. Godfrey et al. found that birth weight was inversely related to carbohydrate intake in early pregnancy [20], which is inconsistent with our results. However, gestational age at the time of nutrient assessment in Godfrey et al.’s study [20] was around 15 weeks gestation, which differed from the present study, making it difficult to conduct a fair comparison. We found that moderate carbohydrate intake was associated with both the total GWG and neonatal birth weight. Godfrey et al. [20] suggested that high carbohydrate intake in early pregnancy suppressed placental growth (and thus fetal growth), as did low dairy protein intake in late pregnancy. However, it was not possible to compare our present results with those of Godfrey et al. because we focused on dietary intake in the second trimester. No significant difference in any background characteristic was apparent between the high and moderate carbohydrate-intake groups. Although statistical significance was not attained, the moderate-intake group contained a higher proportion (54.7%) of wealthier women (monthly household income ≥2000 LKR); this may partially explain the higher GWG and birth weight in this group. The present study showed that babies of rural mothers were heavier than those of urban mothers, although this did not reach the level of significance. This might be attributable to the higher carbohydrate consumption of rural women compared with urban women (Table S1). No direct relationship was observed between second trimester protein intake and neonatal birth weight, which is consistent with results shown by Godfrey et al. [20]. Our univariate analysis showed that women with a total energy intake below the EER delivered neonates with significantly lower mean birth weight compared with women who were above the EER. Although the majority of women in our study were above the EER and RDA for selected macronutrients, individual dietary analysis showed an imbalance in meal habits, for example, a high proportion of carbohydrate and low amount of other important nutrients. In particular, mean fat intake was relatively low. Findings of the current study can be explained by the importance of carbohydrate intake for total GWG, and thereby neonatal birth weight.

Provision of the *Thriposha* supplement for pregnant women aims to maintain satisfactory GWG and reduce the LBW percentage. Although this supplement is distributed free of charge, the present study indicated that the mean *Thriposha* intake was below the recommended intake of 50 g/day. In addition, 11.8% of women reported that they were not consuming the *Thriposha* supplement even though they received it from the community clinics. This may be because the supplement was consumed by the entire family rather than the pregnant woman. However, in the present study, supplement consumption showed no significant effect on either GWG or neonatal birth weight. Further large scale, nationwide studies are recommended to evaluate the effects of supplements distributed free of charge by community clinics.

### 4.1. Limitations

There was a notable data loss between recruitment and the end of data collection, resulting in a smaller sample size than expected. In addition, using a FFQ to collect dietary intake data might have resulted in over/under estimation of actual intake. Despite these limitations, the present study provides the first local estimates of energy and macronutrient consumption among pregnant women in Sri Lanka.

### 4.2. Implications

The Sri Lanka Ministry of Health recommends that the total GWG should be based on the IOM 2009 revised guidelines. Over 15% of women with inadequate GWG delivered LBW infants, indicating that although the IOM guidelines were developed for Americans with larger body frames, they were useful in our setting for identifying women at risk of delivering LBW infants. Culturally and economically competent health care is required to allow Sri Lankan women to achieve a desirable GWG; the emphasis should be on a healthy diet and regular weight monitoring. Nutritional education should be integrated into first-trimester antenatal education sessions, with a focus on meals meeting the dietary requirements of pregnancy and featuring a variety of foods from all food groups. Individual nutritional counseling should be provided to women at risk, especially those with an underweight pre-pregnancy BMI, those exhibiting low GWG, and those on poor diets. We also found that almost all women consumed more than the RDA of carbohydrates during pregnancy. Thus, culturally appropriate dietary recommendations relevant in the Sri Lankan context should be essential components of strategies seeking to prevent low birth weight.

## 5. Conclusions

Second trimester maternal carbohydrate intake shows significant relationships with total GWG and neonatal birth weight. Maintaining a moderate level of carbohydrate intake (430–629 g/day) during the second trimester of pregnancy may promote favorable total GWG and neonatal birth weight in the Sri Lankan context. Our results highlight the need for primary healthcare providers to be vigilant in assessing maternal dietary intake during the second trimester. Individualized education should be provided about good sources of carbohydrates in meals for pregnant women who show weight gain below the recommended levels.

## Figures and Tables

**Table 1:** Participants’ characteristics (*n* = 141).

Variable	Mean (SD)	*n* (%)
Age (years)	28.8 (6.2)	-
Gestational age (weeks) ^a^	38.8 (1.5)	-
Pre-pregnancy weight (kg) ^b^	51.9 (10.2)	-
Pre-pregnancy BMI (kg/m^2^)	22.1 (4.3)	-
Parity		
Primiparous	-	47 (33.3)
Multiparous	-	94 (66.7)
Presence of hyperemesis gravidarum ^c^	-	23 (16.3)
History of miscarriage or abortion	-	38 (26.9)
History of LBW delivery	-	28 (19.9)
Total energy intake (kcal/day)	2921.5 (687.7)	-
Carbohydrate intake (g/day)	532.7 (133.8)	-
Total protein intake (g/day)	71.2 (16.8)	-
Fat intake (g/day)	45.8 (16.9)	-

^a^ *n* = 126. ^b^ Measured at the first antenatal clinic visit, usually around 6–8 weeks gestation. ^c^ During first trimester. BMI: body mass index; LBW: low birth weight. SD: standard deviation.

**Table 2:** Gestational weight gain (GWG) by maternal and neonatal characteristics (ANOVA).

Variable	Sub-category	*n*	GWG up to 28 Weeks (*n* = 105) Mean (SD)	95% CI	*p*-Value	Total GWG (*n* = 119) Mean (SD)	95% CI	*p*-Value
All	-		6.33 (2.85)	5.78–6.89	-	9.30 (3.72)	8.63–9.98	-
Education level	None/primary	16	5.72 (2.80)	4.20–7.24	0.519	7.34 (3.72)	5.51–9.17	0.072
	Secondary	99	6.46 (2.92)	5.85–7.07		9.64 (3.71)	8.90–10.37	
	Higher	2	5.13 (0.11)	1.84–8.42		8.60 (0.57)	3.42–13.78	
Income level (LKR (Sri Lankan rupee))	<9000	5	6.67 (2.57)	3.33–10.00	0.978	9.08 (4.91)	5.75–12.41	0.474
	9000–13,999	20	6.09 (2.62)	4.73–7.46		8.30 (3.44)	6.64–9.96	
	14,000–19,999	32	6.27 (3.35)	5.26–7.27		9.42 (3.23)	8.11–10.74	
	20,000–31,999	45	6.52 (2.77)	5.60–7.45		9.23 (4.21)	8.12–10.34	
	≥32,000	16	6.04 (2.44)	4.30–7.79		10.70 (3.31)	8.77–12.61	
Area of residence	Urban	9	4.53 (3.49)	2.40–6.66	0.167	7.44 (3.93)	4.97–9.92	0.306
	Sub-urban	52	6.68 (3.33)	5.86–7.50		9.28 (3.99)	8.25–10.31	
	Rural	55	6.18 (2.15)	5.37–7.00		9.52 (3.46)	8.52–10.52	
History of LBW deliveries	Yes	22	5.33 (3.03)	4.14–6.52	0.062	8.04 (3.36)	6.48–9.59	0.077
	No	97	6.60 (2.76)	5.99–7.21		9.60 (3.76)	8.85–10.33	
History of miscarriage or abortion	Yes	32	6.47 (2.75)	5.43–7.51	0.759	8.87 (3.75)	7.56–10.17	0.442
	No	87	6.28 (2.91)	5.62–6.94		9.46 (3.77)	8.67–10.25	
Hyperemesis gravidarum ^a^	Yes	20	6.34 (3.21)	5.03–7.65	0.978	9.04 (4.20)	7.37–10.70	0.730
	No	97	6.32 (2.80)	5.70–6.93		9.36 (3.67)	8.60–10.12	
Parity	Primiparous	40	6.89 (2.39)	5.86–7.92	0.208	10.00(4.00)	8.84–11.16	0.146
	Multiparous	79	6.11 (3.00)	5.46–6.76		8.95 (3.54)	8.12–9.77	
Pre-pregnancy BMI category ^b^	Underweight	23	8.43 (2.94) ^1^	7.24–9.62	<0.001 *	12.00(4.07) ^1^	10.58–13.43	<0.001 *
	Normal	69	6.18 (2.26) ^2^	5.51–6.85		9.10 (3.12) ^2^	8.27–9.92	
	Overweight	20	5.73 (3.38) ^2,3^	4.60–6.86		7.89 (3.81) ^2^	6.37–9.42	
	Obese	7	2.76 (0.97) ^3^	0.44–5.08		6.50 (3.30) ^2^	3.92–9.08	
Energy intake ^c^	<EER	21	5.57 (3.04)	4.32–6.82	0.188	8.05 (3.44)	6.44–9.67	0.083
	≥EER	93	6.51 (2.85)	5.87–7.16		9.62 (3.80)	8.86–10.39	
Carbohydrate intake (g/day)	229–429	25	5.97 (3.08)	4.78–7.15	0.798	8.03 (3.60)	6.56–9.50	0.089
	430–629	58	6.41 (2.99)	5.60–7.23		10.00 (3.68)	9.03–10.96	
	630–829	31	6.45 (2.63)	5.29–7.61		9.16 (3.88)	7.84–10.49	
Protein intake ^d^	<RDA	21	5.49 (3.21)	4.24–6.74	0.141	7.81 (3.56)	6.20–9.41	0.039 *
	≥RDA	93	6.53 (2.80)	5.89–7.18		9.68 (3.74)	8.92–10.44	
Sex of the newborn	Male	53	6.44 (2.78)	5.63–7.25	0.552	9.23 (3.31)	8.25–10.21	0.976
	Female	53	6.10 (2.75)	5.28–6.91		9.21 (3.86)	8.23–10.19	

^a^ During first trimester. ^b^ Based on WHO international BMI cut-off values [6]. ^c^ EER for the pregnancy second trimester based on IOM 2009 guidelines [14]. ^d^ RDA based on IOM Food and Nutrition Board 2002/2005 guidelines [15]. ^1,2,3^ Values with the same superscript Arabic numeral do not represent a significance difference. Compared using one-way ANOVA followed by Tukey’s post-hoc test. “n” column represents the subject numbers corresponding to total GWG. * *p* < 0.05. RDA: recommended dietary allowance; WHO: World Health Organization; EER: estimated energy requirement; CI: confidence interval; IOM: Institute of Medicine.

**Table 3:** General linear model for total gestational weight gain (kg).

Variable in Model	Coefficient	95% CI	*t*	*p*-Value
Constant	11.35	8.30–14.39	7.4	<0.001 *
Continuous variables				
Fat intake	−0.04	−0.08–0.00	−1.77	0.08
Categorical variables				
Education (none/primary)—reference level				
Education (secondary)	2.19	0.40–3.97	2.43	0.017 *
Education (higher)	−1.29	−6.24–3.65	−0.52	0.605
Pre-pregnancy BMI category (underweight)—reference level				
Pre-pregnancy BMI category (normal)	−3.84	−5.49–−2.19	−4.62	<0.001 *
Pre-pregnancy BMI category (overweight)	−4.80	−6.96–−2.64	−4.41	<0.001 *
Pre-pregnancy BMI category (obese)	−5.44	−8.49–−2.40	−3.55	0.001 *
Parity (primiparous)—reference level				
Parity (multiparous)	−0.93	−2.27–0.41	−1.38	0.171
Category of carbohydrate intake (229–429 g/day)—reference level				
Category of carbohydrate intake (430–629 g/day)	2.26	0.43–4.08	2.45	0.016 *
Category of carbohydrate intake (630–829 g/day)	1.60	−0.49–3.7	1.52	0.132
Category of protein intake (<RDA)—reference level				
Category of protein intake (≥RDA)	0.42	−1.68–2.52	0.4	0.691

*R^2^* (adjusted) = 16.5%. *n* = 112. * *p* < 0.05.

**Table 4:** Neonatal birth weight by maternal and neonatal characteristics, ANOVA.

Variable	Sub-category	*n*	Birth Weight (g) Mean (SD)	95% CI	*p*-Value
All	-	126	2874.6 (497)	2787.0–2962.2	-
Education level	None/primary	20	2831 (428)	2612.5–3049.5	0.629
	Secondary	100	2867.6 (508)	2769.9–2965.3	
	Higher	4	3091 (391)	2603–3580	
Income level (LKR)	<9000	5	3130 (432)	2702–3558	0.093
	9000–13,999	22	2702 (499)	2498–2906	
	14,000–19,999	31	2965 (573)	2793–3136	
	20,000–31,999	50	2803.3 (454)	2667.9–2938.7	
	≥32,000	16	3036.9 (352.8)	2797.6–3276.2	
Area of residence	Urban	13	2754 (519) ^1,2^	2492–3016	0.011 *
	Sub-urban	54	2747.1 (461.9) ^1^	2618.0–2875.7	
	Rural	57	3011.4 (481.9) ^2^	2886.3–3136.5	
History of LBW deliveries	Yes	24	2684.2 (418.2)	2486.1–2882.2	0.036 *
	No	102	2919.4 (505.2)	2823.3–3015.4	
History of miscarriage or abortion	Yes	33	2898.9 (507.6)	2727.1–3070.8	0.744
	No	93	2865.9 (495.7)	2763.5–2968.3	
Hyperemesis gravidarum ^a^	Yes	20	2800.5 (366.5)	2582.4–3018.6	0.499
	No	104	2882.1 (512.5)	2786.4–2977.7	
Parity	Primiparous	45	2770.1 (430.5)	2624.7–2915.5	0.079
	Multiparous	81	2932.6 (523.9)	2824.2–3041.0	
Pre-pregnancy BMI category ^b^	Underweight	27	2771.5 (459.8)	2523.2–2899.8	0.231
	Normal	70	2912.5 (442.5)	2795.5–3029.5	
	Overweight	22	2895 (673)	2687–3104	
	Obese	7	3059 (464)	2689–3428	
Total GWG category	Within recommended	32	2912.8 (539.7) ^1,2^	2740.8–3084.8	0.042 *
	Below recommended	71	2863.9 (458.5) ^1^	2748.4–2979.3	
	Over recommended	3	3600 (721) ^2^	3038–4162	
Energy intake ^c^	<EER	24	2692 (533)	2491–2892	0.039 *
	≥EER	97	2927.8 (487)	2828.0–2037.6	
Carbohydrate intake (g/day)	229–429	30	2686.7 (498.1) ^1^	2508.5–2864.9	0.033
	430–629	64	2957.7 (486) ^2^	2853.7–3097.7	
	630–829	27	2872.2 (503.6) ^1,2^	2684.4–3060.1	
Protein intake ^d^	<RDA	24	2913 (601)	2708–3117	0.733
	≥RDA	97	2873.1 (479.1)	2771.6–2974.7	
Sex of the newborn	Male	61	2852.9 (477.3)	2726.5–2979.2	0.637
	Female	65	2894.9 (517.7)	2772.5–3017.3	

^a^ During first trimester. ^b^ Based on WHO international BMI cut-off values. ^c^ EER for the pregnancy second trimester based on IOM 2009 guidelines [14]. ^d^ RDA based on IOM Food and Nutrition Board 2002/2005 guidelines [15]. ^1,2,3^ Values with the same superscript Arabic numeral do not represent a significance difference. Sample sizes vary slightly because of missing data. Compared using one-way ANOVA followed by Tukey’s post-hoc test. * *p* < 0.05.

**Table 5:** General linear model for neonatal birth weight (g).

Variable in Model	Coefficient	95% CI	*t*	*p*-Value
Constant	−2011	−4349–327	−1.71	0.091
Continuous variables				
Pre-pregnancy BMI	23.7	3.0–44.4	2.27	0.025 *
Gestational age	102.1	46.7–157.5	3.65	<0.001 *
Fat intake	−2.83	−8.21–2.54	−1.05	0.298
Categorical variables				
Income (<9000 LKR)—reference level				
Income (9000–13,999 LKR)	−209	−679–262	−0.88	0.381
Income (14,000–19,999 LKR)	−43	−495–410	−0.19	0.852
Income (20,000–31,999 LKR)	−74	−523–374	−0.33	0.743
Income (≥32,000 LKR)	156	−322–634	0.65	0.518
Area of residence (urban)—reference level				
Area of residence (sub-urban)	36	−235–308	0.27	0.791
Area of residence (rural)	258	−14–529	1.88	0.062
History of LBW deliveries (yes)—reference level				
History of LBW deliveries (no)	209	−6.0–424.0	1.93	0.057
Parity (primiparous)—reference level				
Parity (multiparous)	187.4	3.2–371.6	2.02	0.046 *
Category of carbohydrate intake (229–429 g/day)—reference level				
Category of carbohydrate intake (430–629 g/day)	312	91–534	2.8	0.006 *
Category of carbohydrate intake (630–829 g/day)	237	−44–517	1.67	0.097
Category of protein intake (<RDA)—reference level				
Category of protein intake (≥RDA)	−66	−326–194	−0.51	0.615

*R^2^* (adjusted) = 13.3%. *n* = 118. * *p* < 0.05.

## Supplementary Materials

The following are available online at www.mdpi.com/2072-6643/9/6/627/s1, Figure S1: Process of data collection, Figure S2: Example estimation of portion sizes for curry dishes (bean curry), Table S1: Second trimester maternal energy and macro-nutrient intake by maternal and neonatal characteristics; ANOVA.
