# Supplementary material for: Impact of Second Trimester Maternal Dietary Intake on Gestational Weight Gain and Neonatal Birth Weight
Source: Nutrients. 2017 Jun 17;9(6):627. doi: 10.3390/nu9060627 (PMC5490606; doi:10.3390/nu9060627)
Supplement: Supplementary file 1 [file nutrients-09-00627-s001.pdf]

Supplementary File

Figure S1. Process of data collection

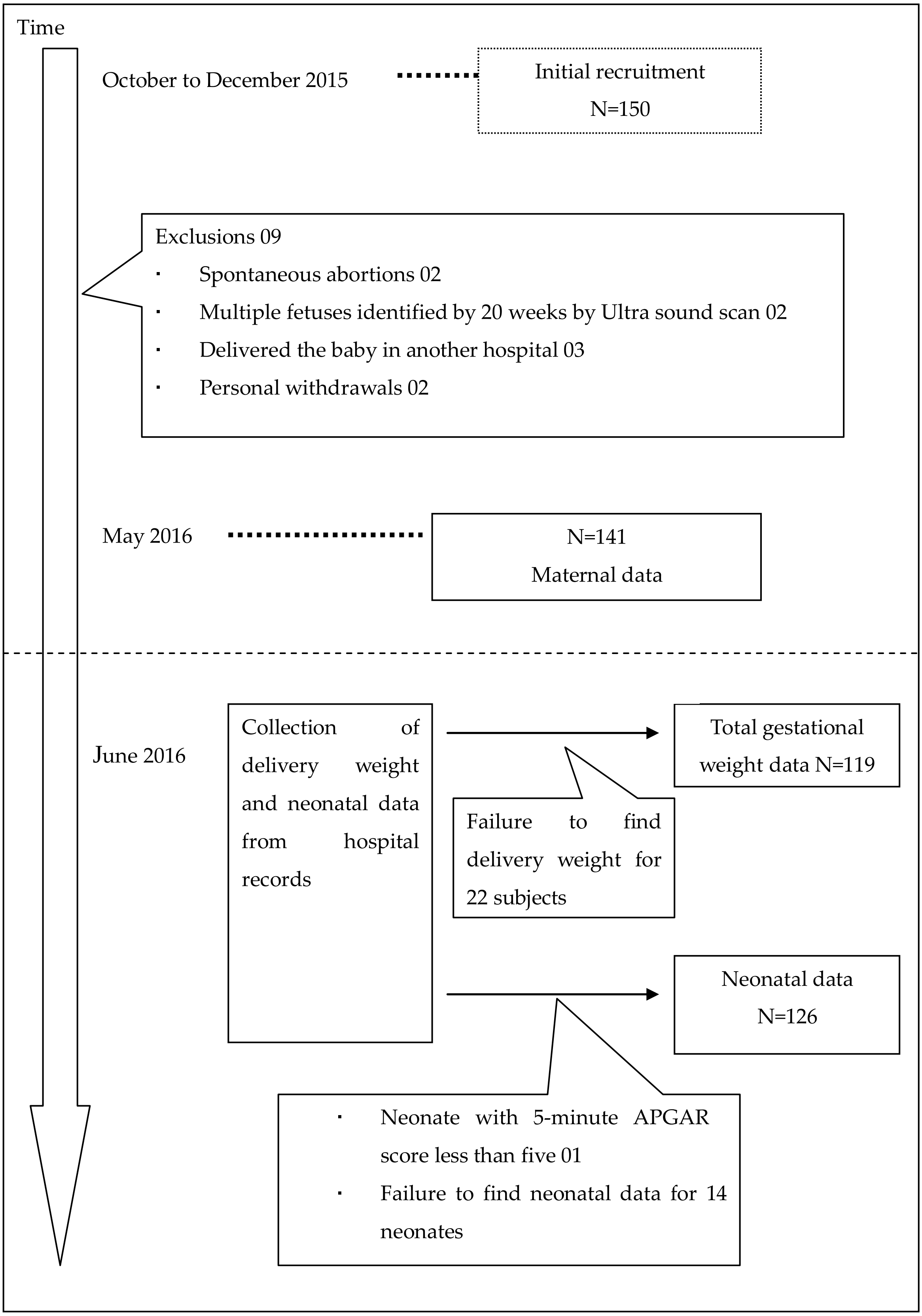

Figure S2. Example estimation of portion sizes for curry dishes (bean curry)

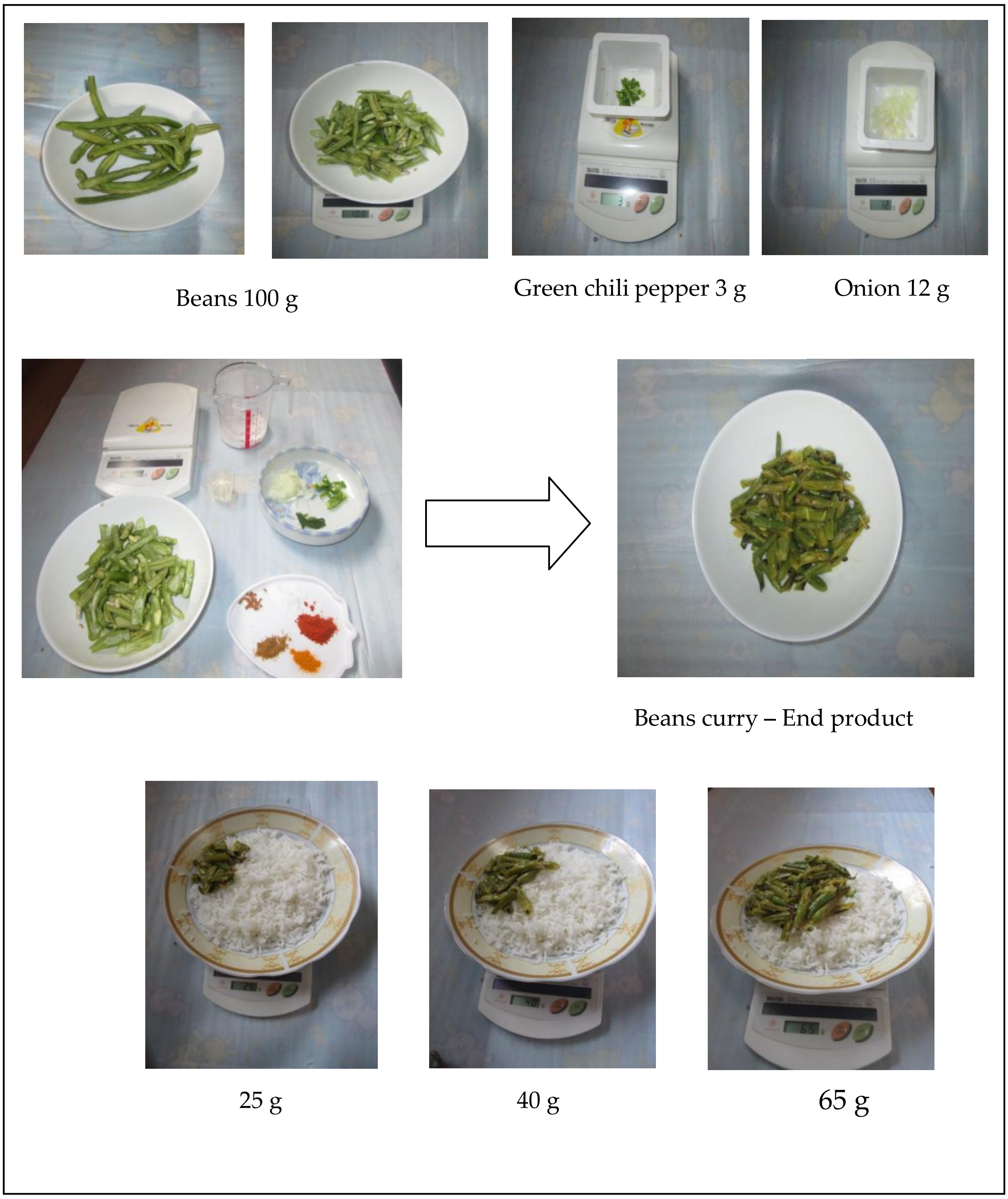

Table S1. Second trimester maternal energy and macro-nutrient intake by maternal and neonatal characteristics ($n = 136$); ANOVA

Variable		Energy Intake		Carbohydrate Intake		Protein Intake		Fat Intake	
		Mean (SD)	p-Value	Mean (SD)	p-Value	Mean (SD)	p-Value	Mean (SD)	p-Value
All		2921.5 (687.7)		532.7 ± 133.8		71.2 (16.8)		45.8 (16.9)	
Level of education									
No/Up to primary	22	2842 (668)	0.804	524.9 (129.9)	0.960	69.0 (17.0)	0.673	40.1 (13.6)	0.287
Secondary	108	2927 (699.9)		533.0 (136.9)		71.3 (16.9)		46.2 (17.3)	
Higher	4	3053 (705)		540.1 (126.3)		74.9 (13.9)		54.8 (18.8)	
Level of monthly household income									
<9000 LKR	4	2608 (555)	0.782	464.1 (147.6)	0.440	68.2 (9.7)	0.870	43.6 (9.9)	0.122
9000-13,999 LKR	22	2940 (624)		549.7 (118.8)		69.3 (15.1)		41.3 (15.0)	
14,000- 19,999 LKR	39	2981 (672)		555.7 (137.7)		70.6 (14.7)		42.4 (14.9)	
20,000-31,999 LKR	51	2856.2 (706.8)		511.5 (130.0)		71.2 (19.8)		47.8 (18.1)	
≥32,000 LKR	18	2993 (819)		531.0 (155.8)		74.8 (18.6)		53.0 (19.0)	
Area of residence									
Urban	13	2781 (718)	0.751	509.2 (134.8)	0.613	68.1 (17.4)	0.767	41.9 (16.1)	0.242
Suburban	59	2906.2 (751)		522.9 (141.4)		71.9 (19.1)		48.3 (19.7)	
Rural	61	2940.9 (626.5)		542.2 (127.8)		70.7 (14.7)		43.8 (14.0)	
History of LBW deliveries									
Yes	28	2875 (726)	0.688	515.7 (142)	0.454	70.7 (17.3)	0.867	48.3 (19.0)	0.384
No	108	2933.6 (680.4)		537.1 (131.9)		71.3 (16.7)		45.2 (6.4)	
History of miscarriage/abortion									
Yes	37	2921 (661)	0.992	532.9 (124.8)	0.991	70.6 (16.6)	0.814	45.8 (17.9)	0.984
No	99	2921.8 (700.7)		532.6 (137.6)		71.4 (17.0)		45.8 (16.7)	
Presence of hyperemesis									
Yes	21	3019 (736)	0.464	539.8 (145.9)	0.771	74 (17.2)	0.401	51.3 (19.0)	0.102
No	113	2898.5 (683.9)		530.4 (133.0)		70.6 (16.9)		44.7 (16.4)	
Parity									
Primiparous	43	3008 (660)	0.320	553.9 (124.0)	0.210	74.2 (17.5)	0.151	44.7 (18.2)	0.595
Multiparous	93	2881.4 (699.8)		522.9 (137.7)		69.8 (16.4)		46.3 (16.4)	
Pre-pregnancy BMI category [a]									
Underweight	27	2920 (671)	0.719	520.1 (127.9)	0.648	72.7 (17.2)	0.889	49.7 (17.5)	0.550
Normal	78	2879 (658.2)		530.7 (132.2)		70.3 (15.3)		44.5 (14.8)	
Overweight	23	3057 (763)		562.6 (139.4)		72.7 (20.4)		46.6 (22.7)	
Obese	8	2775 (873)		508.4 (164.6)		69.8 (21.5)		43.0 (16.7)	
Total gestational weight gain category [b]									
Within	34	3074 (598)	0.644	564.3 (128.4)	0.578	74.8 (13.9)	0.414	46.5 (13.0)	0.551
Below	75	2949.2 (705.4)		535.2 (134.4)		71.8 (17.5)		47.7 (18.7)	
Over	5	2893 (876)		546.7 (175.2)		65.3 (14.9)		39.1 (15.2)	
Birth weight category									
<2500 g	21	2650 (774)	0.192	480.6 (152.3)	0.179	65.6 (17.7)	0.187	47.0 (18.0)	0.389
2500–4000 g	98	2938.1 (648.2)		532.7 (99.1)		72.6 (16.7)		41.7 (13.7)	
>4000 g	2	3088 (756)		600.5 (148.9)		63.4 (17.2)		38.8 (12.7)	

[a] Based on WHO international BMI cut-off values [6]. [b] Based on IOM 2009 re-examine guidelines [14] Compared using one way ANOVA test. Sample sizes for each variable vary slightly bcause of missing data. BMI: Body mass index; LBW: low birth weight; SD: standard deviation; LKR: Sri Lankan rupee.
